# *Clostridium difficile *genotypes other than ribotype 078 that are prevalent among human, animal and environmental isolates

**DOI:** 10.1186/1471-2180-12-48

**Published:** 2012-03-27

**Authors:** Sandra Janezic, Matjaz Ocepek, Valerija Zidaric, Maja Rupnik

**Affiliations:** 1 Institute of Public Health Maribor, Prvomajska 1, 2000 Maribor, Slovenia; 2 Veterinary Faculty, University of Ljubljana, Gerbiceva 60, 1000 Ljubljana, Slovenia; 3 Faculty of Medicine, University of Maribor, Slomskov trg 15, 2000 Maribor, Slovenia; 4 Centre of Excellence, CIPKeBIP, Jamova 39, 1000 Ljubljana, Slovenia

## Abstract

**Background:**

Characterising the overlap of *C. difficile *genotypes in different reservoirs can improve our understanding of possible transmission routes of this pathogen. Most of the studies have focused on a comparison of the PCR ribotype 078 isolated from humans and animals. Here we describe for the first time a comparison of *C. difficile *genotypes isolated during longer time intervals from different sources including humans, animals and the non-hospital environment.

**Results:**

Altogether 786 isolates from time interval 2008-2010 were grouped into 90 PCR ribotypes and eleven of them were shared among all host types and the environment. Ribotypes that were most common in humans were also present in water and different animals (014/020, 002, 029). Interestingly, non-toxigenic isolates were very common in the environment (30.8%) in comparison to humans (6.5%) and animals (7.7%). A high degree of similarity was observed for human and animal isolates with PFGE. In human isolates resistance to erithromycin, clindamycin and moxifloxacin was detected, while all animal isolates were susceptible to all antibiotics tested.

**Conclusion:**

Our results show that many other types in addition to PCR Ribotype 078 are shared between humans and animals and that the most prevalent genotypes in humans have the ability to survive also in the environment and several animal hosts. The genetic relatedness observed with PFGE suggests that transmission of given genotype from one reservoir to the other is likely to occur.

## Background

Intestinal diseases caused by *Clostridium difficile*, mainly after antibiotic treatment, ranges from mild self-limiting diarrhoea to life-threatening pseudomembranous colitis (PMC) and were until recently most commonly seen in hospitalized elderly patients [1]. However, the incidence of community-onset *C. difficile *infection has increased [2-4] and *C. difficile *has also emerged as a pathogen or commensal in different animals such as pigs, calves and chickens [5-7]. Studies on *C. difficile *in the environment are sparse and describe its presence in soil and water [8-11]. For both, environmental contamination and community-associated human infections, animals have been suggested as possible reservoir [5,12,13].

The most prevalent PCR ribotypes differ between humans and food animals. In bovine and porcine hosts PCR ribotype 078 (corresponding to NAP7 and NAP8 by PFGE) is most often detected [14-16]. In humans approximately 300 PCR ribotypes are recognized and the most prevalent in many European countries is PCR ribotype 014/020 (toxinotype 0) [17]. However, in both animals and humans, the distribution of ribotypes is different between countries and from setting to setting, although the heterogeneity is much lower in animals compared to humans. Two large pan-European studies have shown these geographic differences for human-associated *C. difficile *[17,18]. Commonly identified PCR ribotypes for which only regional spreading is suggested are 106, the predominant strain in the UK, ribotype 053 in Austria and 018 which is predominant in Italy [19,20]. In the United States and Canada NAP1, corresponding to PCR ribotype 027 is one of the predominant strains in humans, and in Japan and Korea PCR ribotype 017/toxinotype VIII (A-B+) strain is responsible for CDI outbreaks [21,22].

Most of the comparative studies on *C. difficile *genotypes in humans and food animals have focused on ribotype 078 strain comparisons [23-25]. In addition to being the most frequently isolated strain from pigs and calves in North America and the Netherlands [14-16] it is becoming prevalent in humans in hospitals [17,26] and in the community [3]. It is also often the most prevalent ribotype isolated from food [13,27]. Some other currently important human ribotypes (027, 017) are also reported from animals, [5] but they seem to be less well established in animal hosts. There is currently no published report comparing a large number of strains isolated in the same geographic region from different sources, including humans, animals and the environment. This study makes such a comparison of *C. difficile *strains isolated from three of the possible main reservoirs in a single country to show that ribotypes other than 078 are shared between host types and the environment.

## Results and discussion

### Distribution of PCR ribotypes in different hosts and the environment

**Figure 1:**
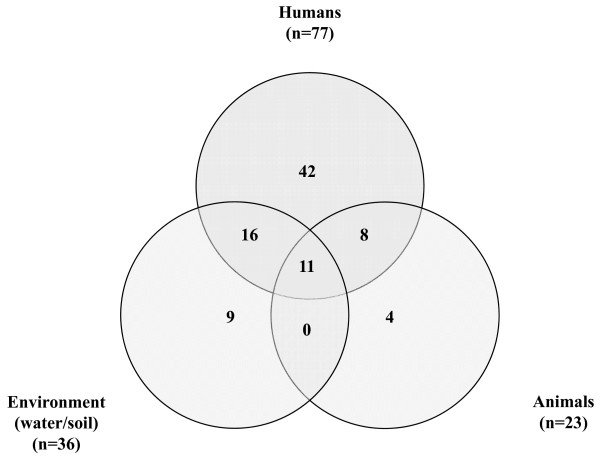
**Comparison of distribution of ribotypes from different reservoirs**.

All 786 isolates that were isolated between 2008 and 2010 were grouped into 90 different PCR ribotypes; human isolates into 77 ribotypes, animal isolates into 23 ribotypes and the environmental isolates into 36 ribotypes (Figure 1, see also Additional file 1: Table S1). There was a considerable overlap between *C. difficile *ribotypes isolated from humans, animals and the environment. Eleven PCR ribotypes were common to all three reservoirs. Sixteen PCR ribotypes were shared only between humans and the environment and were not found in animals, and eight PCR ribotypes were common only to humans and animals. None of the PCR ribotypes identified was shared just between animals and the environment. These results agree in part with previous observations that most genotypes present in animals are also isolated from humans in the same region [15,16,28]. Only a single study compared environmental and human *C. difficile *isolates and also noticed an overlap as 17 of 23 PCR ribotypes were shared between human and environmental strains [9].

The distribution of the most common PCR ribotypes isolated from all three reservoirs in the time period from 2008 to 2010 is shown in Table 1. Interestingly, 30.8% of the environmental isolates were non-toxigenic compared to only 6.5% of human and 7.7% of animal isolates (P < 0.0001; Fisher's exact test). When only toxigenic strains are compared, the two most prevalent PCR ribotypes shared between all three reservoirs were 014/020 and 002 accounting for 20.1% and 8.2% (humans), 24.0% and 23.1% (animals), and 19.8% and 6.2% (environment), respectively. Results for PCR ribotypes 014 and 020 are combined as these two ribotypes have very similar banding pattern which is sometime difficult to distinguish using classical agarose gel-based electrophoresis. Ribotypes 014/020 and 002 are also among the most prevalent ribotypes in Europe [17]. This suggests that ability to survive in different environments plays a role in successful distribution and a high prevalence of a given genotype.

**Table 1:** Most prevalent PCR ribotypes in humans, animals and the environment isolated between 2008 and 2010

PCR ribotype/toxinotype	Humans (n = 601)	Animals (n = 104)	Environment (n = 81)
014/020/0 or I	121 (20.1%)	25 (24.0%)	16 (19.8%)

002/0	49 (8.2%)	24 (23.1%)	5 (6.2%)

001/072/0, tox- or XXIV (CDT+)^§^	42 (7.0%)	8 (7.7%)	2 (2.5%)

012/0	30 (5.0%)	/*	1 (1.2%)

023/IV (CDT+)	30 (5.0%)	/*	3 (3.7%)

018/0	27 (4.5%)	/	2 (2.5%)

029/0	24 (4.0%)	1 (1.0%)	3 (3.7%)

150/0	15 (2.5%)	9 (8.7%)	/

SLO 080/tox-	1 (0.2%)	7 (6.7%)	1 (1.2%)

045/V (CDT+)	1 (0.2%)	5 (4.8%)	/

010/tox-	14 (2.3%)	/*	9 (11.1%)

SLO 057/tox-	1 (0.2%)	/	4 (4.9%)

SLO 064/tox-	2 (0.3%)	/	4 (4.9%)

078/V	6 (1.0%)	/	/

126/V	6 (1.0%)	/	1 (1.2%)

PCR ribotypes marked with* have been found in animals only not between years 2008-10. ^§^Results for PCR ribotypes 001 and 072 are combined in this table since they have a very similar banding pattern which is sometime difficult to distinguish using classical agarose gel-based electrophoresis. Ribotypes 078 and 126 are not among the most prevalent ribotypes and are added only for comparison. (CDT +) - binary toxin positive

As already mentioned, most publications dealing with comparisons of animal and human strains focus on porcine ribotype 078 strains and suggest that pig farms can be an important emerging source of human infection or colonization [23,24]. Our previous studies have shown that ribotype 078 can be completely absent in animals in a given country and that ribotypes other than PCR ribotype 078 (toxinotype V) are prevalent in pigs and other farm animals in Slovenia [7,29,30]. PCR ribotype 078 (toxinotype V) has been found only in humans in Slovenia; of six isolates identified, five came from stool specimens and one from an infected wound. PCR ribotype 126 (toxinotype V and highly related to ribotype 078) has been found in humans (7 isolates) and rivers (1 isolate).

Current epidemic strain, PCR ribotype 027/toxinotypeIII/NAP1 was reported in domestic animals and their environment mostly in Canadian studies [16,31,32]. Our collection did not include any animal 027 strain. First human PCR ribotype 027 strain was identified only in 2010 and this type accounted for as little as 2.7% (16/601) of all human isolates (see Additional file 1: Table S1).

### Characterisation of most common PCR ribotypes found in animals and humans

Due to the large number of isolates available (n = 1078) only a subset of representative strains from the most common PCR ribotypes found in humans and animals were further characterized with PFGE and antimicrobial susceptibility testing. Selected strains belonged to 7 different PCR ribotypes: 014/020/(toxinotype 0), 010/(non-toxigenic strain; tox-), SLO 055/(tox-), 023/(toxinotype IV), 029/(toxinotype 0), 002/(toxinotype 0) and 150/(toxinotype 0). A single strain of PCR ribotype SLO 055 was included in the comparison as its PCR ribotyping profile is very similar to the profile of PCR ribotype 010.

**Figure 2:**
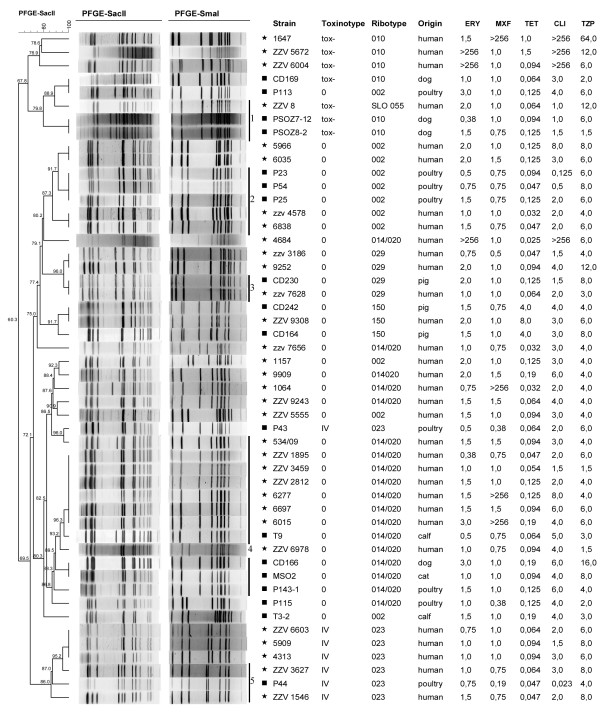
**PFGE dendrogram of *Sac*II restriction digest**. PFGE dendrogram (*Sac*II restriction digest) and the association with PFGE patterns of *Sma*I restriction digest, toxinotype, PCR ribotype, origin and antibiotic susceptibility testing. The dendrogram is coded according to origin; human isolates (*) and animal isolates (■). The MICs are given in terms of mg/L. The bars represent the groups (1-5) of human and animal isolates having identical *Sma*I and/or *Sac*II banding pattern.

The majority of strains of a single PCR ribotype isolated from humans and animals grouped together with PFGE regardless of which restriction enzyme was used (*Sma*I or *Sac*II). With *Sma*I groups were more coherent and in four toxigenic PCR ribotypes (002, 029, 014/020 and 023), human and animal isolates had indistinguishable banding pattern (groups 2-5 on the Figure 2). However, when restriction was performed with *Sac*II, only one pig isolate had an identical banding pattern to the human one while other animal isolates differed from human isolates of the same ribotype but still belonged to the same pulsotype (defined by 80% and 85% similarity for *Sma*I and *Sac*II, respectively). Within non-toxigenic group of strains (group 1 on the Figure 2) a human isolate of PCR ribotype SLO 055 (related to ribotype 010) had an identical banding pattern when restriction was performed with *Sma*I, though with *Sac*II the human and the two animal isolates belonged to different pulsotypes. These results are in agreement with previous studies reporting human and food animal strains to be very closely related or indistinguishable using different typing methods. In the USA toxinotype V strains (PFGE type NAP7/NAP8 corresponding to ribotype 078) isolated from humans and pigs have been found to be indistinguishable with PFGE [25]. In more recent study by Koene *et al*. (2011), comparing human and animal isolates from the same geographic location and time period with the MLVA, authors confirmed previously observed relatedness between human and porcine ribotype 078 strains but in contrast to our PFGE results (group 4 on Figure 2) no genetic relatedness could be observed for human and animal isolates of ribotype 014 and 012 [24,33].

A great focus has been given on pigs as a source of human CDI. Poultry which can harbour a variety of human associated PCR ribotypes has been so far overlooked [7]. Two human and one poultry isolate of ribotype 023 (toxinotype IV, binary toxin positive) had indistinguishable banding pattern with *Sma*I and belonged to the same pulsotype with *Sac*II (group 5 on Figure 2). For companion animals (dogs and cats) has also been shown to harbour the same ribotypes as humans [15,33]. In our study, one dog and one cat isolate of PCR ribotype 014/020 had identical banding pattern as the human isolates of the same PCR ribotype using *Sma*I restriction enzyme and belonged to the same pulsotype when *Sac*II restriction patterns were compared (group 4 on Figure 2).

The genetic relatedness of human and animal isolates shown in this study suggests that not only ribotype 078 strains show zoonotic potential. Other ribotypes are shared between animals and humans as well, and that alongside porcine and cattle, poultry can also be an important link for human CDI. Whether and how often the transmission from animals to humans and/or vice versa occurs have yet to be determined.

Table 2 lists the range of MICs of the most common PCR ribotypes isolated from humans and animals for five out of six antibiotics tested. All isolates tested were fully susceptible to rifampicin. With a few exceptions all strains within a single PCR ribotype had similar but not identical MICs for all antibiotics tested. Exceptions include high MICs to erythromycin (ERY), clindamycin (CLI) and moxifloxacin (MXF) (Table 2, Figure 2) for human ribotype 014/020 strains. Interestingly, all three human ribotype 010 strains (all non-toxigenic) had MICs ≥ 256 mg/ml for CLI and ERY (2 isolates), and CLI plus MXF (1 isolate). This multiple drug resistance in non-toxigenic strains could suggest that these strains might serve as reservoir of antibiotic resistance determinants. Strains resistant to the antibiotics tested were found only among human isolates. However, only for moxifloxacin, MICs for human isolates were more likely to be above the MIC_50 _of all isolates tested (P < 0.05) (Table 3).

**Table 2:** MIC ranges of most common PCR ribotypes isolated from humans and animals

PCR ribotype	ERY (mg/L)	MXF (mg/L)	TET (mg/L)	CLI (mg/L)	TZP (mg/L)
002 (n = 11)	0.5-3	0.75-1.5	0.032-0.19	0.125-8	3-8

023 (n = 7)	0.5-1.5	0.19-1	0.047-0.094	0.023-3	4-8

029 (n = 4)	0.75-2	0.5-1	0.047-0.125	1.5-4	3-12

014/020 (n = 18)	0.38- > 256	0.38- > 256	0.025-0.19	1.5- > 256	1.5-16

010 (n = 6)	0.38- > 256	0.75- > 256	0.064-1.5	1- > 256	1.5-64

150 (n = 3)	1.5-2	0.75-1	4-8	3-8	4-8

ERY - erythromycin; CLI - clindamycin; TET- tetracycline; TZP - piperacillin/tazobactam; MXF - moxifloxacin; Ribotype SLO 055 (n = 1) is not included in this table, but is included in Table 3

**Table 3:** MIC_50/90 _values of human and animal *C.difficile *isolates

Host		ERY (mg/L)	MXF (mg/L)	TET (mg/L)	CLI (mg/L)	TZP (mg/L)
Humans (n = 32)	MIC_50_	1.5	1	0.094	3	6

	MIC_90_	3	> 256	0.19	> 256	12

	Range	0.38- > 256	0.50- > 256	0.025-8	1- > 256	1.5-64

Animals (n = 18)	MIC_50_	1	0.75	0.125	3	6

	MIC_90_	2	1	0.19	5	8

	Range	0.38-3	0.19-1	0.047-4	0.023-6	1.5-16

All (n = 50)	MIC_50_	1.5	1	0.094	3	6

	MIC_90_	3	1.5	0,19	8	8

	Range	0.38- > 256	0.19- > 256	0.025-8	0.023- > 256	1.5-64

## Conclusions

Ribotype 078 is not the only ribotype significantly shared between humans and animals. Here we show that all genotypes that are among most prevalent in (hospitalized) humans have a tendency to prevail also in animals and in the environment (river water) and that a better environmental survival might be part of their virulence spectrum. Human and animal isolates of the same PCR ribotype clustered together with PFGE and had mostly also similar MIC values for all antibiotics tested. This genetic relatedness suggests that transmission of given genotype from one reservoir to the other is likely to occur.

## Materials and methods

### *C. difficile *isolates

Isolates included in the comparison originated from humans, animals and the non-hospital environment and are part of the strain collection at the Institute of Public Health Maribor. Altogether 1078 isolates from Slovenia were available. Isolates from all three reservoirs were sampled from the overlapping geographical locations and time periods.

Human isolates (n = 690) were recovered by routine diagnostic laboratories throughout Slovenia and submitted to our laboratory for typing between 2006 and 2010. The isolates were from hospitalized patients and from patient from other institutions (less than 1% of all isolates), and were not submitted as a part of an outbreak investigation.

Environmental isolates were from river water (n = 77) and soil (n = 4), and were isolated between 2008 and 2010. River water isolates from 17 rivers throughout Slovenia were collected as a part of the national surveillance of surface waters. Soil isolates originated from the field near the poultry farm from which poultry samples were collected. The isolates were cultured as described elsewhere [11].

Animal isolates (n = 307) were from piglets (n = 138), calves (n = 6), a horse (n = 1), poultry and birds (n = 150), and dogs and cats (n = 12) isolated between 2006 and 2010. Piglet isolates (including symptomatic and asymptomatic animals) were from 9 pig farms located in different parts of Slovenia. Poultry isolates were from two big facility for laying hens and three smaller farms. Dog, cat and calf isolates were from different Slovenian households and farms. Stool samples or rectal swabs collected from these animals were processed as described elsewhere [7,29]. Due to the clustering (i.e. large number of isolates from the same animal farm), only 156 animal isolates (piglets (n = 16), poultry and birds (n = 121), dogs and cats (n = 12), calves (n = 6) and a horse) were included in the final analysis (only a single strain isolated per sampling and per farm).

The final number of isolates included in the comparison of prevalence and distribution of PCR ribotypes was 786 (601 from human, 104 from animals and 81 from the environment) from the time period 2008-10, as for this time period environmental strains were available. For the PFGE and antimicrobial susceptibility testing of human and animal strains, 50 isolates from broader time interval (2006-2010) were selected.

### Molecular characterisation

All isolates were characterised by toxinotyping and PCR ribotyping. Toxinotyping involved amplification and subsequent restriction of PCR fragment A3 (part of *tcd*A) and B1 (part of *tcd*B). PaLoc negative strains were confirmed by amplification of a 115 bp-long insert with primers Lok1/Lok3 [34]. The binary toxin gene (*cdt*B) was detected as described previously [35].

PCR ribotyping was performed with the primers 16S (5'-GTGCGGCTGGATCACCTCCT) and 23S (5'-CCCTGCACCCTTAATAACTTGACC) as described by Bidet *et al*. (1999) [36]. After amplification PCR products were concentrated to a final volume of 25 μl by heating at 75°C for 45 min before electrophoresis in 3% agarose gel (Bio-Rad, USA) in 1× TAE buffer for 5 h at 2.5 V/cm. BioNumerics software (Applied Maths, Belgium) version 6.10 was used to analyze the banding patterns. PCR ribotypes for which reference strains were available were designated by standard Cardiff nomenclature (002, 029...; 46 Cardiff type strains were available in our laboratory for comparisons) while others were designated by internal nomenclature (SLO and 3-digit code).

A total of 50 *C. difficile *isolates of the most prevalent PCR ribotypes found in humans and animals isolated between 2006 and 2010 were further analyzed with PFGE and antimicrobial susceptibility testing. Selection of the strains was made by randomly selecting human and animal strains isolated in the same time period and from the same geographic locations covering different Slovenian regions. These included 32 human isolates and 18 animal isolates from pigs (n = 3), poultry (n = 8), a cat (n = 1), calves (n = 2) and dogs (n = 4).

### Pulsed field gel electrophoresis

PFGE was performed as described elsewhere [37]. Genomic DNA was digested with 15 U of *Sac*II or *Sma*I (New England BioLabs, UK) overnight and Biometra PFGE System (Biometra, Germany) was used for electrophoresis. Dendrograms were constructed using BioNumerics software 6.10 (Applied Maths, Belgium) by the UPGMA clustering method, using the Dice coefficient with position tolerance and optimization of 1.10%. Clusters with ≥ 80% (*Sma*I) or ≥ 85% (*Sac*II) similarity were considered to be distinct pulsotypes.

### Antimicrobial susceptibility testing

The same strains typed by PFGE were also tested for antibiotic resistance. Minimum inhibitory concentrations (MICs) of 6 antimicrobial agents; rifampicin (RIF), moxifloxacin (MXF), erythromycin (ERY), piperacilin/tazobactam (TZP), tetracycline (TET) and clindamycin CLI), were determined by the E-test method. An inoculum of McFarland 1.0 was swabbed on Brucella blood agar supplemented with haemin (5 μg/mL) and vitamin K_1 _(1 μg/ml). Plates were incubated for 48 h at 37°C in an anaerobic atmosphere. *Bacteroides thetaiotaomicron *ATCC 29741 was used as a quality control strain. Resistance was defined according the following breakpoints established by the CLSI guidelines: clindamycin (CLI) ≥ 8 mg/l, tetracycline (TET) ≥ 16 mg/l, piperacillin/tazobactam (TZP) ≥ 128 mg/l, moxifloxacin (MXF) ≥ 8 mg/l, erythromycin (ERY) ≥ 8 mg/l and rifampicin (RIF) ≥ 4 mg/l [38,39]. MIC_50 _and MIC_90 _were calculated for human and animal isolates. The frequencies at which the MICs for human isolates were above the MIC_50 _and MIC_90 _values for all isolates tested were compared with Fisher's exact *t *test.

## Authors' contributions

SJ carried out the molecular typing, performed data analysis, participated in the design of the study and helped to draft the manuscript. VZ carried out microbiological work and in part molecular typing of animal and environmental isolates. MO participated in microbiological work on animal isolates. MR participated in design of the study and coordination and helped to draft the manuscript. All authors have read and approved the final manuscript.

## Supplementary Material

Additional file 1 **Table S1**. PCR ribotypes identified in humans, animals and the environment between 2008 and 2010 in Slovenia. Click here for file
