# Supplementary material for: Clostridium difficile genotypes other than ribotype 078 that are prevalent among human, animal and environmental isolates
Source: BMC Microbiol. 2012 Mar 27;12:48. doi: 10.1186/1471-2180-12-48 (PMC3353227; doi:10.1186/1471-2180-12-48)
Supplement: Additional file 1 — Table S1. PCR ribotypes identified in humans, animals and the environment between 2008 and 2010 in Slovenia. [file 1471-2180-12-48-S1.PDF]

PCR ribotype/toxinotype	Humans<br>n = 601	Animals<br>n = 104	Environment<br>n = 81
014/020/0 or I	121 (20,1 %)	25 (24 %)	16 (19,8 %)
002/0	49 (8,2 %)	24 (23,1 %)	5 (6,2 %)
072 (0,tox-; XXIV - CDT+)	33 (5,5 %)	3 (2,9 %)	1 (1,2 %)
023/IV (CDT+)	30 (5 %)	/	3 (3,7 %)
012/0	30 (5 %)	/	1 (1,2 %)
018/0	27 (4,5 %)	/	2 (2,5 %)
029/0	24 (4 %)	1 (1 %)	3 (3,7 %)
011/049/0	18 (3 %)	4 (3,8 %)	2 (2,5 %)
070/0	17 (2,8 %)	3 (2,9 %)	/
027/III (CDT+)	16 (2,7 %)	/	/
056/XII/I	16 (2,7 %)	1 (1 %)	1 (1,2 %)
015/0	15 (2,5 %)	/	1 (1,2 %)
150/0	15 (2,5 %)	9 (8,7 %)	/
010/tox-	14 (2,3 %)	/	9 (11,1 %)
081/0	11 (1,8 %)	/	/
003/0	10 (1,7 %)	1 (1 %)	/
001/0	9 (1,5 %)	5 (4,8 %)	1 (1,2 %)
005/0	9 (1,5 %)	3 (2,9 %)	3 (3,7 %)
046/0	8 (1,3 %)	/	1 (1,2 %)
SLO 036/0	7 (1,2 %)	/	/
SLO 083/I	7 (1,2 %)	/	/
078/V (CDT+)	6 (1 %)	/	/
087/0	6 (1 %)	/	/
126/V (CDT+)	6 (1 %)	/	1 (1,2 %)
SLO 002/tox-	6 (1 %)	/	1 (1,2 %)
103/0	5 (0,8 %)	3 (2,9 %)	/
017/VIII	4 (0,7 %)	/	/
SLO 074/XII	4 (0,7 %)	/	/
SLO 079/IX (CDT+)	4 (0,7 %)	/	/
SLO 084/tox-	4 (0,7 %)	/	1 (1,2 %)
SLO 055/tox-	3 (0,5 %)	/	/
SLO 096/0	3 (0,5 %)	/	/
SLO 110/0	3 (0,5 %)	/	/
131/XXIV (CDT+)	3 (0,5 %)	2 (1,9 %)	/
SLO 069/0	3 (0,5 %)	1 (1 %)	1 (1,2 %)
SLO 017/VIII	2 (0,3 %)	/	/
SLO 022/0	2 (0,3 %)	/	/
SLO 028/tox-	2 (0,3 %)	/	/
SLO 054/0	2 (0,3 %)	/	/
SLO 075/0	2 (0,3 %)	/	/
SLO 076/0	2 (0,3 %)	/	/
SLO 064/tox-	2 (0,3 %)	/	4 (4,9 %)
SLO 025/0	2 (0,3 %)	/	2 (2,5 %)
SLO 063/0	2 (0,3 %)	/	1 (1,2 %)
SLO 034/0	2 (0,3 %)	1 (1 %)	/
SLO 082/IX or XXI(CDT+)	2 (0,3 %)	1 (1 %)	/
SLO 049/III (CDT+)	2 (0,3 %)	1 (1 %)	1 (1,2 %)
SLO 120/0	2 (0,3 %)	/	/

**PCR ribotype/toxinotype**	**Humans n = 601**	**Animals n = 104**	**Environment n = 81**
033/XIa (CDT+)	1 (0,2 %)	/	/
053/0	1 (0,2 %)	/	/
106/0	1 (0,2 %)	/	/
SLO 030/tox-	1 (0,2 %)	/	/
SLO 038/tox-	1 (0,2 %)	/	/
SLO 058/0	1 (0,2 %)	/	/
SLO 065/0	1 (0,2 %)	/	/
SLO 066/0	1 (0,2 %)	/	/
SLO 068/0	1 (0,2 %)	/	/
SLO 072/0	1 (0,2 %)	/	/
SLO 073/0	1 (0,2 %)	/	/
SLO 077/tox-	1 (0,2 %)	/	/
SLO 086/0	1 (0,2 %)	/	/
SLO 111/tox-	1 (0,2 %)	/	/
SLO 112/0	1 (0,2 %)	/	/
SLO 116/0	1 (0,2 %)	/	/
SLO 117/0	1 (0,2 %)	/	/
SLO 057/tox-	1 (0,2 %)	/	4 (4,9 %)
SLO 071/0	1 (0,2 %)	/	2 (2,5 %)
SLO 081/0	1 (0,2 %)	/	1 (1,2 %)
SLO 085/0	1 (0,2 %)	/	1 (1,2 %)
SLO 080/tox-	1 (0,2 %)	7 (6,7 %)	1 (1,2 %)
045/V (CDT+)	1 (0,2 %)	5 (4,8 %)	/
SLO 067/tox-	1 (0,2 %)	/	/
SLO 053/0	1 (0,2 %)	/	/
SLO 118/0	1 (0,2 %)	/	/
SLO 119/III (CDT+)	1 (0,2 %)	/	/
SLO 125/0	1 (0,2 %)	/	/
SLO 126/IX (CDT+)	1 (0,2 %)	/	/
SLO 012/tox-	/	/	2 (2,5 %)
SLO 093/0	/	/	2 (2,5 %)
SLO 089/0	/	/	1 (1,2 %)
SLO 091/tox-	/	/	1 (1,2 %)
SLO 092/tox-	/	/	1 (1,2 %)
SLO 094/0	/	/	1 (1,2 %)
SLO 095/tox-	/	/	1 (1,2 %)
SLO 113/0	/	/	1 (1,2 %)
SLO 045/0 or tox-	/	1 (1 %)	/
SLO 060/XI (CDT+)	/	1 (1 %)	/
SLO 114/0	/	1 (1 %)	/
SLO 090/0	/	1 (1 %)	/
SLO 144/V/CDT+	/	/	1 (1,2 %)
